# Unlocking Allelic Diversity for Sustainable Development of Salinity Stress Tolerance in Rice

**DOI:** 10.2174/1389202922666211005121412

**Published:** 2021-12-30

**Authors:** Rishiraj Raghuvanshi, Ashish Kumar Srivastava, Satish Verulkar, Penna Suprasanna

**Affiliations:** 1 Department of Plant Molecular Biology and Biotechnology, Indira Gandhi Krishi Vishwavidyalaya, Raipur-492012, India;; 2 Nuclear Agriculture and Biotechnology Division, Bhabha Atomic Research Centre, Mumbai-400085, India;; 3 Homi Bhabha National Institute, Mumbai-400094, India

**Keywords:** Allelic diversity, salt tolerance, QTLs, transporters, rice, *OsHKT1;1*, *OsHKT1;5*

## Abstract

Rice is a major cereal crop, negatively impacted by soil-salinity, both in terms of plant growth as well as productivity. Salinity tolerant rice varieties have been developed using conventional breeding approaches, however, there has been limited success which is primarily due to the complexity of the trait, low yield, variable salt stress response and availability of genetic resources. Furthermore, the narrow genetic base is a hindrance for further improvement of the rice varieties. Therefore, there is a greater need to screen available donor germplasm in rice for salinity tolerance related genes and traits. In this regard, genomics based techniques are useful for exploring new gene resources and QTLs. In rice, the vast allelic diversity existing in the wild and cultivated germplasm needs to be explored for improving salt tolerance. In the present review, we provide an overview of the allelic diversity in the Quantitative Trait Loci (QTLs) like Saltol, qGR6.2, qSE3 and RNC4 as well as genes like *OsHKT1;1*, *SKC1* (*OsHKT1;5/HKT8*) and *OsSTL1* (salt tolerance level 1 gene) related to salt tolerance in rice. We have also discussed approaches for developing salt-tolerant cultivars by utilizing the effective QTLs or genes/alleles in rice.

## 1 INTRODUCTION

Rice is an important staple cereal food crop consumed by half of the world population [1], and almost ~90% of it is produced and consumed in Asian countries [2]. Soil and water salinity are the major constraints for rice production, especially in the South and Southeast Asia [3] and in the areas where farmers use mostly artificial irrigation with low quality of water and poor drainage system [4]. Being a salt sensitive crop, rice suffers mortality when Electrical Conductivity (EC) reaches ≥10 dSm-^1^ [5]. All the plant developmental stages are considered to be salt sensitive, however, the effect of salinity during the reproductive stage maximally reduces the seed yield up to 50% [6]. Salt stress induces multiple plant processes, such as reduction in K^+^ uptake, photosynthetic rate, osmotic adjustment, antioxidant enzymatic activity and disturbed plant-water homeostasis [7-10]. Salt tolerance is a complex polygenic trait regulated through several pathways and efforts for improving salt tolerance depend on insightful details of the molecular mechanisms [11]. Different classes of transporters, including *NHX* (Na^+^/H^+^ antiporter), *HKT* (high affinity K^+^ transporter), *CHX* (Cation-H^+^ exchanger), *SOS1* (Salt Overly Sensitive-1), and *NSCC* (Non-Selective Cation Channel) are involved in mitigating salt stress by reducing Na^+^ concentration in the cytosol [12]. The perception of Na^+^ ion is mediated by non-selective cation channels of depolarization type [13], which trigger the cytosolic Ca^2+^ transients. Subsequently, SOS (Salt overly sensitive) pathway and several MAP kinases are activated to control downstream signaling. The SOS signaling pathway constitutes *SOS1, SOS2* and *SOS3* proteins. The cytosolic Ca^2+^ interacts with *SOS3* to activate *SOS2* (serine/threonine kinase protein) and, ultimately, *SOS1* is activated to pump Na^+^ out of the cells. In addition, *SOS2* is also involved in Na^+^ sequestration into vacuoles. Recently, a membrane located glycosyl inositol phosphorylceramide has also been discovered, which regulates SOS pathway in plants [14]. Several researchers have studied the isolation and characterization of different Na^+^/H^+^ antiporter genes and their overexpression for enhancing salinity stress tolerance [15, 16]. Among them, the *HKT* gene family in rice contributes significantly to Na^+^ exclusion [17], and especially, *OsHKT1;5* is an important player detected in the underlying region of Saltol, which is one of the salt tolerance-specific quantitative trait loci (QTL) [18, 19].

Molecular marker(s) have been successfully applied for the improvement of salt tolerance in several genetic backgrounds, such as naturally diverse lines, wild types, bi-parental population and landraces of rice [20]. QTL is a genetic locus that correlates with variation of a trait in the phenotype of a population. QTLomics is the study to identify alleles or genes of various quantitative traits in crop plants [21]. Recent advances in sequencing technology and statistical solutions have enabled the analysis of plant genomes through genome-wide association studies (GWAS) or linkage disequilibrium (LD) mapping. These approaches have accelerated the process of identification of causal genetic variants and the subsequent characterization of complex genetic traits with an extremely high resolution map than linkage mapping [22]. GWAS detects allelic variants for complex traits and provides high density single nucleotide polymorphism (SNP) marker data in a given population. Common alleles show their frequencies of more than 1% in the population or frequent enough that these can be queried by genotyping in standard marker panels [23] whereas, the frequency of rare alleles with less than 1% in the population can be explored with the help of sequencing techniques [24-26]. There are several studies on QTL mapping and haplotype diversity for salinity stress tolerance in crop plants. QTLs and markers flanking the QTLs for salinity tolerance have been exploited in breeding and rice improvement programs. Progress in this area has emphasized that there is a greater need for the identification genes to be used as promising candidates/haplotypes for salt tolerance at a specific developmental stage. In the present review, we have presented a detailed account of the allelic diversity for salt tolerance in rice germplasm and discussed the QTLs discovery, candidate gene-based association study and GWAS with some success stories.

## 2 QTLs DISCOVERY USING MOLECULAR MARKERS

In the past few decades, conventional breeding methods have been employed for the improvement of salt tolerance, but there are limitations, such as long breeding time, yield penalty and polygenic complexity of the trait [27]. Rice exhibits vast genetic diversity through the availability of elite germplasm, wild species and landraces, such as Pokkali, which show salt tolerant characteristics [28, 29]. Precise and efficient selection of salt tolerance traits has become possible due to the availability of phenomics and molecular markers and improvement strategies such as marker-assisted selection (MAS), which is a useful tool for the transfer QTLs or gene(s) for enhancing salt-tolerance in rice (Table **1**).

**Fig. (1):**
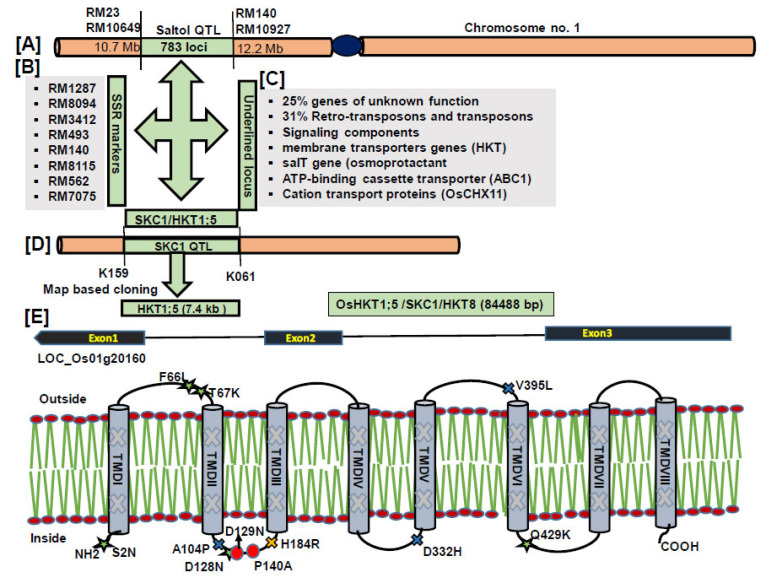
**Saltol QTL for regulating Na^+^/K^+^ ratio and salt tolerance in rice**. Saltol QTL located on chromosome no.1 [34, 43]. [**A**] QTL linked SSR markers [**B**] QTL underlined Locus [**C**] map-based cloning of SKC1 QTL encoding HKT1;5 gene [**D**] and *OsHKT1;5* /SKC1 model [**E**] with 8 transmembrane domain (TMD) based on hydrophobicity plot analysis. Amino acid substitutions are shown on helix in blue cross, black asterisks and red circle as reported by Ren *et al*. [48] in between Nona Bokra and Koshihikari, Yang *et al*. [84] in 529 accessions and Negrao *et al*. [70] in 392 germplasms, respectively. Orange color cross indicate SNP showing strong correlation. (*A higher resolution / colour version of this figure is available in the electronic copy of the article*).

Mapping studies have played a great role in the identification of various QTLs associated with tolerance to different abiotic stresses in rice [30-42]. Saltol one of the most important QTLs, was identified in a cross between Pokkali (salinity stress tolerant genotype) and IR29 (salt susceptible genotype). Plants harboring Saltol exhibited low Na^+^ uptake, high K^+^, and low Na^+^ to K^+^ ratio in shoots under salt stress conditions [43]. Amplified fragment length polymorphism (AFLP) markers have also been used for mapping Saltol using recombinant inbred lines (RILs). Among these, one line (FL478 RIL), identified as the most salt tolerant line, is currently being used for marker assisted breeding programs. Bonilla *et al*. [34] reported that Saltol, between RM23 and RM140 markers (~10.7-12.2 mb), contained ~783 loci. Most of the Saltol associated loci encode for transcription factors and membrane transporters genes, while 25% have unknown function [44, 45]. This Saltol genomic region also contain specific genes for salt tolerance, including *OsHKT1;5* (a Na^+^/K^+^ co-transporters gene) [18, 19], *saIT* gene (osmoprotactant) [46], high affinity K^+^ transporter 1 (*HKT1*), ATP-binding cassette transporter (*ABC1*) [4] and cation transport proteins (*OsCHX11*) [47] (Fig. **1**). Similarly, eight QTLs for variation in K^+^ and Na^+^ levels were detected in F_2_ segregating population from a cross between Nona Bokra (Indica salt tolerant variety) and Koshihikari (susceptible japonica variety) [31]. Of these, two important QTLs like qSNC-7 and qSKC-1/SKC1 accounted for 48.5 and 40.1% phenotypic variance in Na^+^ and K^+^ levels in shoot, respectively. Ren *et al*. [48] investigated the role of SKC1 in K^+^ homeostasis in the salt tolerant genotype. The results from the map based cloning using 2,973 back cross F_2_ (BC_2_F_2_) population showed that *SKC1* locus was ~7.4 kb located between K036 and Pr, having an ORF which showed substantial similarity with High affinity K^+^ (HKT) transporters and corresponding to *OsHKT8*, also referred as *OsHKT1;5* [49]. These results suggest that *OsHKT1;5* is a key player for regulating K^+^/Na^+^ homeostasis under salt stress conditions (Fig. **1**).

Several studies have also been conducted to transfer Saltol into cultivated genetic backgrounds. For example, Saltol has been successfully transferred into salt susceptible Indian variety Pusa basmati-1 from the highly salt tolerant, donor line FL478. The near isogenic lines (NILs) showed seedling stage tolerance with all agronomic attributes of Pusa basmati-1 [50]. The study resulted in the detection of three QTLs linked SSR markers RM8094, RM493, and RM10793 [50]. In another study, using a RILs population developed from Liang-You-Pei-Jiu (Super hybrid rice) and PA64s, an important QTL, qSL7 was identified for shoot length on chromosome 7. Fine mapping of QTL region (252.9 kb) resulted in 40 genes, out of which one gene was found to code for DNA binding domain protein for salinity stress tolerance [51]. Krishnamurthy *et al*. [52] performed marker assisted backcross breeding to transfer Saltol from FL478 (highly salt tolerant RIL from Pokkali) to salt susceptible, high yielding varieties, Pusa44 and Sarjoo52. The transfer of QTL was facilitated by recombinant selection with flanking SSR markers RM493 and G11a, and the presence of QTL was confirmed by foreground selection after each back cross with RM3412 and AP3206 markers. Another study involving cultivars, Pusa44 and Sarjoo52 showed a difference in haplotype from donor line FL478 at four markers loci in the Saltol region *i.e*., RM3412, RM493, AP3206 and G11A. Out of these, RM3412 and AP3206 were found to be tightly linked markers, whereas G11A and RM493 were useful as recombinant markers. A study on screening of six polymorphic SSR markers (closely linked to Saltol QTL) resulted in the identification of RM10843 with the highest allelic number whereas, RM140 and RM10748 were identified with the lowest allelic number, and the polymorphism information content (PIC) ranged from 0.1780 for RM10748 to 0.7659 for other marker alleles [53]. PIC is often used as a tool to detect polymorphisms, and has been used in selecting markers for genetic studies. Huong *et al*. [54] reported the highest polymorphism with 0.58 PIC value with SSR markers RM237, RM10748, and RM224.

In a study on breeding for salinity tolerance using Bangladeshi rice landraces, eight Sequence Tagged Site (STS) markers were designed for *SKC1, DST,* and *SalT* genes [55], of which Wn11463 (a STS marker for *SKC1*) and RM22418 were found to be associated with salinity stress tolerance. Another specific QTL qGR6.2 was also mapped for seed germination under salinity stress in the indica landraces [56]. This was confirmed by fine mapping with BC_2_F_2_ population and qGR6.2 (approx 66 kb) was found to be flanked by the Z654 and Z619 markers, which have been very useful in MAS program for improving germination stage salinity stress tolerance [56]. A related QTL qST1.1 was also identified for seedling stage salt tolerance, flanked by SSR marker RM8904 and RM493 in F_2_ population derived from indica sea rice 86 (seedling salt tolerant genotype) and Dianjingyou 1 [57].

Molecular markers associated with QTLs affecting important salt tolerant traits have also been useful as indirect selection criteria to improve salinity stress tolerance traits. The use of MAS for improving simple agronomic traits has been successfully adopted in crop breeding programmes. However, research on MAS for improving complex traits such as drought and salinity stress tolerance has only begun in the last decade. Unlike the situation with simple/qualitative traits where marker information has been frequently and successfully utilized in breeding programmes, the situation is different with complex/quantitative traits, such as salinity stress tolerance which is controlled by multiple genes and has low heritability and strong genotype to environmental interactions (GxE). There is a greater need for studies on the introgression salt tolerant QTLs in different genetic backgrounds and environments for use in rice-breeding programs [58].

## 3 CANDIDATE GENE-BASED ASSOCIATION STUDY

**Fig. (2):**
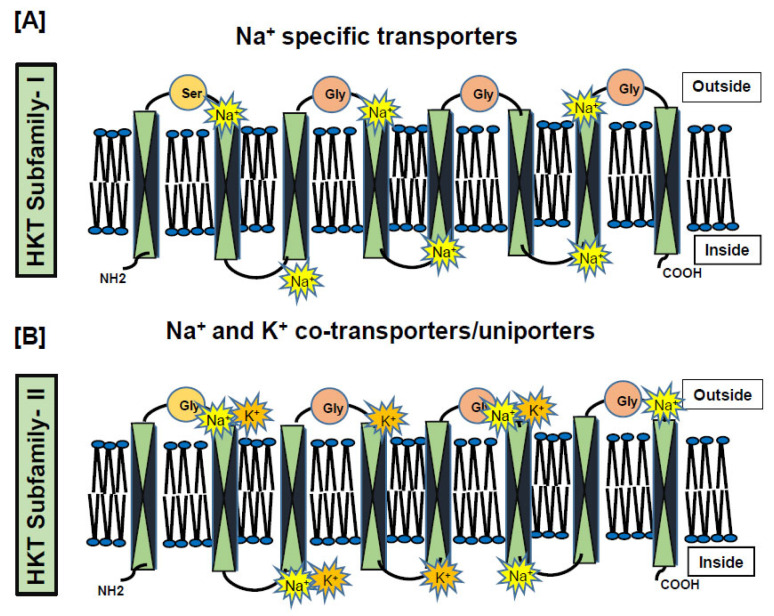
**Classification of HKT family and transport characteristics.** HKT family divided into two subfamilies based on amino acid sequence similarity and transport features [61, 62]. [**A**] subfamily-I codes for Na^+^ specific transporters, containing Ser-Gly-Gly-Gly signature motif (*OsHKT1;1*, OsHKT1;2, OsHKT1;3, OsHKT1;4 and *OsHKT1;5*) While subfamily-II [**B**] codes for Na^+^ and K^+^ co-transporters or Na^+^-K^+^ uniporters (OsHKT2;1, OsHKT2;2, OsHKT2;3 and OsHKT2;4) with Gly-Gly-Gly-Gly signature [63] and serine/glycine residue determined the selectivity of ions. (*A higher resolution / colour version of this figure is available in the electronic copy of the article*).

Salinity stress imposes osmotic and ionic effects on plants, which alter plant growth and productivity. In addition, salt stress imposes secondary effects, such as nutrient imbalances, induces oxidative stresses and hormonal imbalances [7]. To counteract such adverse effects, plants respond in different ways through mechanisms of ion exclusion or ion homoeostasis. The allelic variation in genes for ion homeostasis has been investigated with the help of candidate gene-based association study (CGAS). CGAS is usually performed in the genetic variants existing within the pre-specified genes and traits of interest. The rationale behind this approach is that the allelic variation present within the genes may constitute few mutations which will directly affect the function or alter the phenotype. CGAS is also helpful when none of the genome wide associations are found to be significant for a trait of interest. The, gene-based association studies have been used for the mining of several alleles for salinity stress tolerance in rice (Table **2**). The most prominent ones include members from *HKT* gene family, which encode trans-membrane channel proteins. Dicots such as Arabidopsis contain only one *HKT* gene, known as *AtHKT1;1,* whereas monocots plants have multiple *HKT* genes [17]. For example, rice has eight HKT gene members [59, 60], which are categorized into two subfamilies on the basis of amino acid sequence similarity and transport characteristics (Fig. **2**) [61, 62]. Subfamily-1 is comprised of *OsHKT1;1, OsHKT1;2, OsHKT1;3, OsHKT1;4* and *OsHKT1;5*, which are Na^+^ specific transporters having Ser-Gly-Gly-Gly signature motif whereas, subfamily-2 includes *OsHKT2;1, OsHKT2;2, OsHKT2;3* and *OsHKT2;4* that functions as Na^+^ and K^+^ co-transporters or Na^+^-K^+^ uniporters with Gly-Gly-Gly-Gly signature motif [63]. Oomen *et al*. [64] identified a new isoform of *OsHKT2;2/1* (referred to as *No-OsHKT2;2/1*) from Nona Bokra, which showed strong permeability to Na^+^ and K^+^ at high concentration of salt [64].

In general, the selectivity of Na^+^ - selective channels like HKTs is associated with a serine residue whereas replacing it with glycine leads into infiltration of multiple cations. (Fig. **2**) [19]. In some plants, for example rice, eucalyptus and Thellungiella, *HKT2;1* is shown to contain Ser-Gly-Gly-Gly motif but it is described as subfamily-II protein having permeability for both Na^+^ and K^+^ [65]. In wheat, structural studies of *TmHKT1;5-A* and *TaHKT1;5-D* showed mutations in amino acid residues responsible for transport activity and improved salinity by the exclusion of Na^+^ [66]. These studies suggest that OsHKT1;5 is one of the key genes for salinity stress tolerance, which exhibits significant functional diversity [49]. It is also suggestive that the determination of Na^+^ and K^+^ selectivity is not completely based on specific filter motif and that other structural components may also contribute to the salinity tolerance trait. QTLs reported for class 1 *HKT* gene family have generated a wealth of information on the regulatory mechanism of uptake of Na^+^ in maize and rice [67, 68]. The specific variant of QTL, SKC1 on chromosome no 1 was functionally characterized for maintaining shoot K^+^ content under salinity stress conditions in the salt-tolerant rice genotype (Nona Bokra) but not in the salt susceptible genotype.

In plants, allele mining is used to detect superior alleles within the related germplasm, which may have had mutations during the course of evolution. Once the alleles showing superior performance are identified, they can be utilized to develop allele specific markers for use in marker assisted selection [67]. First study on allele mining was conducted in the Indian wild rice accessions for *HKT* gene family [67]. Among HKT members, *OsHKT2;3* showed the highest and *OsHKT1;1,* the lowest nucleotide and haplotype diversity. Haplotype analysis also indicated substantial amount of natural variation, particularly for *OsHKT1;5* and *OsHKT2;3* genes among the Indian wild rice (Table **2**). Two important alleles, haplotypes H5 and H1 of *OsHKT1;5* and *OsHKT2;3* respectively, were found to be associated with salinity stress tolerance suggesting that these alleles are good candidates for introgression into high yielding cultivars. In another study, nucleotide and haplotype diversity study was performed with 21 salinity stress responsive genes [69] to reveal variation in the transporter gene family. Significant association of SNPs with salt tolerance genes like *BADH2, HsfC1B, MIPS1, MIPS2, MYB2, NHX1, NHX2, NHX3, P5CS1, P5CS2, PIP1, SIK1, SOS1*, and *SOS2* genes was observed. Ren *et al*. [48] reported six SNPs in the OsHKT1;5 coding sequence, which showed four amino acid changes (Fig. **1**; A104P, H184R, D332H, V395L). These changes were further validated by Negrao *et al*. [70] and Shohan *et al*. [49]. Among these four alterations, two substitutions, working synchronously, namely, leucine to valine (V395L) in position 395 and histidine to aspartate (D332H) in position 332 were noted only in the tolerant genotypes, including a distant halophyte, with an important function in salinity tolerance. The results further explained the adaptive genetic variation for stress tolerance and that the minor haplotypes may have recently evolved, denoting a recent expansion of Indian wild rice genotypes.

Using Eco-TILLING approach, studies have been conducted to identify gene variants in salt responsive genes related to Na^+^/K^+^ ratio, signaling pathways and osmo-protection. Negrao *et al*. [70] genotyped 392 rice germplasms and found that 40 allelic variants and 11 SNPs were significantly associated with salt tolerance. Further, the consequence of associated SNPs was evaluated at the protein level with the help of bioinformatics tools. Three non-synonymous SNPs were found in *OsHKT1;5* with significant associations, out of which two SNPs were observed with two residual differences between ‘Nipponbare genotype’ (haplotype A) and IR29 (haplotype B), specifically D129N and P140A (Fig. **1**). The authors also reported two substitutions of which, T67K mutation could destabilize one of the transmembrane domain in OsHKT1;5 and the P140A mutation could change phosphorylation efficiency [70]. Similarly, screening of 550 genotypes of *Oryza sativa* from diverse geographical locations indicated that there were seven major alleles of *OsHKT1;5* within *Oryza sativa*; while, three minor alleles were identified within the Japonica, aromatic and IR29 lines [71]. The foregoing account suggests the association of natural genetic variations in the salt responsive candidate genes belonging to different gene families with salt tolerance phenotype and their haplotype variation in different geographic regions. The *OsHKT1;5,* a member of *HKT* gene family was found as a major key regulating gene that mediates Na^+^ exclusion in the vasculature to protect leaf blades and reproductive tissues in rice [72] and wheat [73]. In addition, candidate gene wise association studies also emphasize that *OsHKT1;5* should be included, as one of the key candidates, in the screening and development of salt-tolerant genotypes.

## 4 GENOME WIDE ASSOCIATION-BASED STUDY

Genome-wide association study (GWAS) or linkage disequilibrium mapping is one of the potential molecular breeding tools used to detect the association between genetic variants, characterized at the whole genome and for a particular trait of interest. Several GWAS studies have been performed in the last decade to explore the allelic variations associated with salt-tolerance in rice (Supplementary Table **1**). Using a collection of 235 temperate japonica rice genotypes, Frouin *et al*. [74] identified 50 QTLs and 300 candidate genes associated with mild salinity stress tolerance at the seedling stage. Most of the QTLs were found to be closely related to calcium signaling and metabolism related genes [74]. Another QTL, qSE3 was discovered for seed germination stage and seedling establishment using map-based cloning method [75]. The results revealed that qSE3 encodes for a K^+^ transporter, *HAK21,* which significantly enhanced K^+^ and ABA at the germination stage under salt stress. In GWAS panel comprising of 208 mini-core rice accessions from 25 countries, thirteen traits associated with salt tolerance (ST) were investigated at the germination and seedling stages. The results revealed 20 quantitative trait nucleotides (QTN) for thirteen traits for salinity stress tolerance (Supplementary Table **1**) [76]. Three salt tolerance genes were found in the QTN underline region (*SKC1 (HKT1;5), OsTZF1* and *OsEATB* for QTN, *qSNK1 qSST5* and *qSST9).* Apart from *HKT1;5,* two other salt responsive genes were also detected, encoding for a transporter and a putative protein of the kinase family. In another GWAS, 104 Thai rice accessions were screened using different photosynthetic parameters, cell membrane stability and yield-related traits. Based on the observations of net photosynthetic rate under salt stress, the data revealed 200 loci containing 448 SNPs. The top four regions with a high number of significant SNPs were found on chromosomes no 1, 2, 8 and 12 [77].

Besides the genetic control of the nuclear genome on environmental stress tolerance, cytoplasmic genome also plays a key role in the adaptation mechanisms in crop plants. The effect of cytoplasmic genome on QTL model has been investigated for leaf K^+^ concentration and grain number in the reciprocal population derived from Horkuch (salt tolerant genotype) and IR29 (a high yielding cultivar) [78]. The results showed a significant cytoplasmic effect on salt tolerance associated traits suggesting that the cytoplasmic effect could be related to plastid symporter activity and their interaction with nuclear genes. GWAS of 390 indica and japonica genotypes under moderate (9 dS_m-1) salinity stress showed that the Japonica genotypes had lower root Na^+^ concentration than Indica ones [79]. There was a strong association between root Na^+^ / K^+^ ratio and root Na^+^ concentration, located in a region of chromosome 4 (~575 Kb), which was named as Root Na^+^ Content 4 (RNC4). Further analysis of this locus revealed *OsHKT1;1* and *OsHKT1;4* genes as the possible candidates. Three non-synonymous SNPs were also detected in *OsHKT1;1* with high frequency rate in the Indica subpopulation, which was validated by introducing *OsHKT1;1* from indica variety into japonica background. Another nine SNPs identified in the study were found to have nine amino acid substitutions in the coding sequence of *OsHKT1;5* [80]. Out of nine, H284R was reported as a highly significant substitution in the coding region of *OsHKT1;5* genes. The results suggested that apart from Saltol and SKC1, RNC4 can be considered as important QTLs for the regulation of shoot and root Na^+^ concentration in rice [79]. The study has also opened up new genetic resource for modifying Na^+^ levels in crop plants (Supplementary Table **1**).

Wild germplasm is a treasure of valuable gene resources for biotic and abiotic stress tolerance for introgression into cultivated germplasm. Quan *et al*. [81] isolated a salt tolerant line DJ15 from a cross between Dongxiang (salinity tolerant wild cultivar) and Ningjing16 (cultivated variety) [81] and, sequence analysis of variants in the QTL underlying region revealed few transporters genes (*OsSKC1/OsHKT8/OsHKT1;5* and *OsHAK6*). One of the RILs was found to be tolerant and contained two different QTLs (qST1.2 and qST6), suggesting that the use of two or more QTLs for introgression can be a useful strategy to develop a salt tolerant variety. In another study, the role of *MADS31* gene belonging to the MADS-box family transcription factor was validated in a GWAS study with 295 genotypes for salinity stress tolerance at the germination stage [82]. Recently, two more salt tolerant genes *OsSTL1*, (salt tolerance level 1) and *OsSTL2* (salt tolerance level 2), were identified in the 3000 Rice Genome Project (3K-RG) [83]. The results revealed the *OsSTL1* gene to be a homolog of salt tolerance gene SRP1 (Stress associated RNA binding protein-1) of Arabidopsis. Another GWAS for salt tolerance was performed to evaluate a large population using 2255 markers for different phenotypic traits [82-84]. Several candidate genes were identified, out of which, 43 genes were found to regulate salt tolerance. The study also found five most important genes (*MYB6, GAMYB, HKT1;4, CTR3,* and *SUT1*) and two new genes (LOC_Os02g49700 and LOC_Os03g28300) suggesting that such a gene resource could be used in the screening of salt tolerant genotypes. Thus, a substantial progress has been made in GWAS for the discovery of a number of QTL/gene in rice [90-94], which can be further characterized using functional genomics approach. Most of the QTLs have been mapped for Na and K content in the leaf tissue because of the predominance of tolerance mechanism of salt exclusion in leaves of tolerant genotypes. Although these traits are essentially independent, none of the known salt tolerant landraces have more than a few salt tolerance gene resources, while considerable variation in the extent of expression of those genes exists among different genotypes, suggesting the likelihood of identifying even better donors and alleles of useful genes. Gene pyramiding of major QTLs and genes for contributing traits at seedling and reproductive stages is needed for developing resilient salt-tolerant cultivars.

## CONCLUSION

In conclusion, several salt tolerance related genes/QTLs with considerable allelic diversity have been discovered in rice. These include saltol QTL (for Na^+^/K^+^ ratio and seedling stage salt-tolerance), qSNC-7 (for shoot Na^+^ accumulation) and qSKC-1 (for shoot K^+^ level). The fine mapping of QTLs has also revealed molecular alterations in *OsHKT1;5,* which directly influence Na^+^/K^+^ homeostasis. The candidate gene-based approach and GWAS have highlighted the role of key SNPs in salt tolerance associated genes like MADS-box and MYB family transcription factor, vacuolar cation/proton exchanger, *OST3/OST6* family protein and *HKT1;1/1;5*. Most of the identified germplasm with salt tolerant characteristics have been identified within the related wild or feral species that could not be utilized in breeding programmes without inherent difficulties. The advent of molecular markers and mapping technology has helped in the identification of genes or QTLs of interest for complex traits, such as salinity stress tolerance and to transfer them from un-adapted genetic backgrounds into commercial varieties *via* MAS. Despite significant progress in unraveling the underlying mechanisms, many highly effective QTLs/genes/allele might be cultivar-specific, and hence further research is necessary on the adaptability over a wide range of cultivars and genetic backgrounds. Thus, the potential of allelic diversity can be unleashed for application in gene pyramiding, and introgression of effective QTLs for improving salt tolerance in high yielding rice cultivars.

## Figures and Tables

**Table 1:** QTLs and linked markers reported for salt tolerance in different rice varieties.

**QTL**	**Chromosome**	**Traits**	**Flanking Markers**	**Parents**	**References**
*SKC1*	1	Shoot K^+^ concentration	C1211-S2139 (Os01g0307500)	Nona Bokra and Koshihikari	[31, 48]
*Saltol*	1	Shoot Na^+^ concentration, Shoot K^+^ concentration and Ratio of Na^+^ to K^+^ concentrations in shoots	P3/M9-8-P1/M9-3	Pokkali and IR29	[43]
	1		RM23-RM140	Pokkali and IR29	[34]
	1		RM1287-RM10825	Pokkali and IR29	
-	1	Leaf Na^+^ concentration, leaf K^+^ concentration, Leaf K^+^/Na^+^ ratio	RG811-RZ276	Co39 and Moroberekan	[85]
*qNaSH-1.1*, *qKSH-1.1*, *qNa/KSH-1.1*	1	Shoot Na^+^ concentration, Shoot K^+^ concentration and Ratio of Na^+^ to K^+^ concentrations in shoots	RM294-HvSSR01-46	CSR27 and MI48	[86]
-	1	Na^+^ uptake, shoot K^+^ concentration and ratio of Na^+^ to K^+^ concentrations in shoots	-	IR4630 and IR15324	[87]
*qSKC-1*, *qSNC-1*	1	Shoot Na^+^ concentration, shoot K^+^ concentration	RM580-RM9	Changbai10 and Dongnong425	[88]
qSDS-1	1	Survival days of seedling	C813-C86	Nona Bokra andKoshihikari	[48]
qSDS-6	6		C214-R2549	Nona Bokra andKoshihikari	[48]
qSDS-7	7		R2401-L538T7	Nona Bokra andKoshihikari	[48]
qSNC-7	7	Shoot Na^+^ concentration	C1057-R2401	Nona Bokra andKoshihikari	[48]
qSNTQ-7	7	Shoot Na^+^ total quantity	C1057-R2401	Nona Bokra andKoshihikari	[48]
qSKC-1	1	Shoot K^+^ concentration	C1211-S2139	Nona Bokra andKoshihikari	[48]
qRNC-9	9	Root Na^+^ concentration	R1751-R2638	Nona Bokra andKoshihikari	[48]
qRNTQ-1	1	Root Na^+^ total quantity	C813-C86	Nona Bokra andKoshihikari	[48]
qRKC-4	4	Root K^+^ concentration	C891-C513	Nona Bokra andKoshihikari	[48]
qRKC-7	7		C1057-R2401	Nona Bokra andKoshihikari	[48]
qRKTQ-7	7	Root K^+^ total quantity	C1057-R2401	Nona Bokra andKoshihikari	[48]
*qSKC-1*	1	Shoot K^+^ concentration	IM7685-IM9146	‘Nipp’ × ‘ZYQ8’	[89]
*qSNC-1*	1	Shoot K^+^ concentration	RM283-IM9146		[89]
*qSKC-1*	1	Shoot K^+^ concentration	IM7685-IM9146	*rss4* × ‘ZYQ8’	[89]
*qSNC-1*	1	Shoot K^+^ concentration	RM283-IM9146		[89]

**Table 2:** SNPs and haplotypes reported in candidate gene-based association for salt tolerance in rice.

**GENES**	**MSU. ID**	**Chromosome**	**Function**	**No. of SNPs**		**No. of haplotypes**	**References**
				**Coding**	**Non- coding**		
*HKT1;1*	LOC_Os04g51820	4	Na^+^ specific transporters (subfamily 1)	8	3	13	[67]
*HKT1;2*	Chr4:30548314..30545885	1	Na^+^ specific transporters (subfamily 1)	0	20	12	[67]
*HKT1;3*	LOC_Os02g07830	2	Na^+^ specific transporters (subfamily 1)	8	6	10	[67]
*HKT1;4*	LOC_Os04g51830	4	Na^+^ specific transporters (subfamily 1)	2	42	20	[67]
*HKT1;5*	LOC_Os01g20160	1	Na^+^-K^+^ cotransporters or Na^+^-K^+^ uniporters (subfamily 2)	8	37	23	[67]
*HKT2;1*	LOC_Os06g48810	6	Na^+^-K^+^ co-transporters or Na^+^-K^+^ uniporters (subfamily 2)	5	46	18	[67]
*HKT2;3*	LOC_Os01g34850	1	Na^+^-K^+^ cotransporters or Na^+^-K^+^ uniporters (subfamily 2)	24	4	23	[67]
*HKT2;4*	LOC_Os06g48800	1	Na^+^-K^+^ cotransporters or Na^+^-K^+^ uniporters (subfamily 2)	0	4	5	[67]
*HKT1;1*	LOC_Os04g51820	4	Na^+^ specific transporters (subfamily 1)	N/A	N/A	2	[73]
*HKT1;5*	LOC_Os01g20160	1	Na^+^-K^+^ cotransporters or Na^+^-K^+^ uniporters (subfamily 2)	N/A	N/A	7	[73]
*SNAC*	LOC_Os01g66120.1	1	NAC domain containing protein	N/A	N/A	2	[73]
*DUF6*	LOC_Os01g10990	1	Integral membrane protein	N/A	N/A	8	[73]
*CCC*	LOC_Os08g23440	8	cation chloride co-transporter	N/A	N/A	3	[73]
*SOS1*	LOC_Os12g44360	12	Na^+^/H^+^Exchanger	N/A	N/A	9	[73]
*OsHKTI;5*	LOC_Os01g20160	1	Na^+^-K^+^ cotransporters or Na^+^-K^+^ uniporters (subfamily 2)	29	57	17	[70]
*OsNHX1*	LOC_Os07g47100	1	Vacuolar Na^+^/H^+^ antiporter, salt tolerance	10	54	13	[70]
*SalT*	LOC_Os01g24710	1	Jacalin-like lectin domain containing protein	13	109	13	[70]
*OsRMC*	LOC_Os04g56430	4	PF01657 AT4G23180.1 CRK10,RLK4 cysteine-rich RLK (RECEPTOR-like protein kinase) 10	3	3	4	[70]
*HKT1;1*	LOC_Os04g51820	4	Na^+^-specific transporters (subfamily 1)	N/A	N/A	N/A	[73]
*HKT1;4*	LOC_Os04g51830	4	Na^+^-specific transporters (subfamily 1)	N/A	N/A	N/A	[73]
